# Biomechanical and tomographic differences in the microarchitecture and strength of trabecular and cortical bone in the early stage of male osteoporosis

**DOI:** 10.1371/journal.pone.0219718

**Published:** 2019-08-08

**Authors:** Poh-Shiow Yeh, Yuan-Wen Lee, Wei-Hui Chang, Weu Wang, Jaw-Lin Wang, Shing-Hwa Liu, Ruei-Ming Chen

**Affiliations:** 1 Department of Neurology, Chi Mei Medical Center, Tainan, Taiwan; 2 Graduate Institute of Medical Sciences, College of Medicine, Taipei Medical University, Taipei, Taiwan; 3 Department of Anesthesiology, School of Medicine, College of Medicine, Taipei Medical University, Taipei, Taiwan; 4 Anesthesiology and Health Policy Research Center and Department of Anesthesiology, Taipei Medical University Hospital, Taipei, Taiwan; 5 Division of General Surgery, Department of Surgery, Taipei Medical University Hospital, Taipei, Taiwan; 6 Institute of Biomedical Engineering, National Taiwan University, Taipei, Taiwan; 7 Institute of Toxicology, College of Medicine, National Taiwan University, Taipei, Taiwan; 8 Cell Physiology and Molecular Image Research Center, Wan Fang Hospital, Taipei Medical University, Taipei, Taiwan; Indiana University Purdue University at Indianapolis, UNITED STATES

## Abstract

Osteoporosis is a continuous process of loss of bone tissue. Compared to women, osteoporosis in men is associated with greater morbidity and mortality. In this study, we conducted tomographic and biomechanical evaluations of trabecular and cortical bone in the early stage of male osteoporosis. Male Wistar rats were subjected to orchiectomy and sham operation. Four weeks after being castrated, decreased levels of testosterone in plasma were found and resulted in concurrent bone loss. Separately, the orchiectomy led to significant tomographic alterations in the trabecular bone number, trabecular separation, and trabecular pattern factor. Data of a mechanistic compression test further showed that the orchiectomy diminished the maximum loading force, displacement at maximum load, energy at maximum load, and ultimate stress. Interestingly, orchiectomy-triggered changes in the maximum loading force and tomographic parameters were highly correlated. In contrast, tomographic and biomechanical analyses showed that 4 weeks after rats were orchiectomized, the thickness, area, maximum loading force, bone stiffness, energy at maximum load, and ultimate stress of the cortical bone were not changed. Taken together, this study showed specific differences in the microarchitecture and strength of trabecular bone in the early stage of male osteoporosis.

## Introduction

Osteoporosis, a systemic aging skeletal disorder, is characterized by increased bone fragility and high risks of bone fracture [1, 2]. According to a report by the World Health Organization, osteoporosis is one of the most common metabolic diseases in the world [3]. Thus, osteoporosis is a public and critical healthcare issue now and in the future. Multiple risk factors are involved in the incidence of osteoporosis, including age, genetics, hormonal variations, smoking, and calcium and vitamin D deficiencies [4]. In the past, osteoporosis was generally considered to be a woman's disease, because men have larger skeletons, bone loss starts later and advances more slowly, and there was no period of rapid hormonal change [5, 6]. Recently, osteoporosis in men has been a focus of researchers, healthcare professionals, and clinicians [7]. Compared to women, osteoporosis in men exhibits different pathophysiological stages. Riggs et al. showed that loss of cortical bone begins after the age of 75 years in men [8]. In women, loss of cortical bone occurs much earlier [9]. According to a statistical report by the National Osteoporosis Foundation, men experience 42% of total lifetime trabecular bone loss before the age of 50 years [5]. In women, trabecular bone is lost with age, and such a bone injury is accelerated during perimenopause [10]. Nevertheless, older men are more likely to become osteoporotic with a total testosterone deficiency [11, 12]. As a result, osteoporosis in men and women has noteworthy variances in pathophysiology and clinical risks.

Bone loss in men commonly comes about later in life. A variety of hazard factors such as chronic diseases, lifestyle habits, medication use, male sex hormones, calcium intake, heredity, and aging contribute to causes of osteoporosis in men [13]. Among these factors, the sex hormones, androgens, play crucial roles in regulating bone remodeling and maintaining the bone mass [14]. In particular, testosterone has the highest concentration and mainly controls the development and maintenance of masculine physiognomy [12, 15]. Levels of blood androgens decrease with aging, and an androgen deficiency is recognized as the main cause of osteoporosis in men [14, 16]. In elderly men, preserving ideal levels of androgens is essential to preventing osteoporosis and its complications [12]. In addition, bone fractures are major complication of osteoporotic patients [17]. Prominently, osteoporosis in men has a higher mortality rate due to hip, vertebral, and other major fractures [18, 19]. High morbidity and mortality of osteoporosis-connected fractures impose heavy economic burdens on patients, their families, and society.

Dual-energy x-ray absorptiometry (DXA), micro-computed tomography (μCT), and peripheral quantitative computed tomography (pQCT) are widely used and powerful tools for analyses of bone geometry and densitometry [20]. These radiographic methodologies can evaluate the bone architecture and bone mass by measuring x-ray absorptiometry of mineral tissues [20, 21]. In the clinic, DXA is a standard approach to assess bone mineral density (BMD) through scanning and recording the mineral absorption content of the target site. Nevertheless, previous studies showed that BMD cannot completely and accurately reflect the risk of osteoporosis-associated bone fractures [21–23]. Especially in elderly men, only 21% of non-vertebral fracture patients were detected as having osteoporosis, defined as a BMD of < -2.5. Thus, analysis of BMD is insufficient to elucidate the bone strength and fracture risk [22]. Biomechanical approaches can provide alternative methodologies for assessing bone strength and osteoporosis-associated fracture risks. Mechanistic reduced-platen compression (RPC) and four-point bending (FPB) tests can be applied to measure the strength of trabecular and cortical bone [24, 25]. Ovariectomy and orchiectomy are two typical surgical techniques for induction of bone loss in female and male rodents, respectively [26]. Our previous study showed that ovariectomy led do a significant reduction in levels of serum estrogen and a consequent loss of bone mass in female rats [27]. In comparison, orchiectomy can induce testosterone deficiency and bone loss in male animals via the receptor activator of nuclear factor kappa β-ligand (RANKL)-osteoprotegerin (OPG) signaling pathway [26, 28, 29]. In this study, we used tomographic and biomechanical approaches to evaluate differences in the microarchitecture and strength of trabecular and cortical bone in the early stage of male osteoporosis using orchiectomy rats as the experimental model. We hypothesize that orchiectomy can cause associated declines in the microarchitecture and strength of trabecular and cortical bone in the early stage of osteoporosis in rats.

## Material and methods

### Animals

Male Wistar rats (250~300 g) purchased from BioLASCO (Taipei, Taiwan) were housed in an environmentally controlled room, with a standard 12-h light/dark cycle and free access to laboratory chow (Teklad Global Diets, Indianapolis, IN, USA) and tap water. All procedures were performed according to the National Institutes of Health *Guidelines for the Use of Laboratory Animals* and were approved by the Institutional Animal Care and Use Committee of Taipei Medical University-Wan Fang Hospital, Taipei, Taiwan (Approval no. WAN-LAC-103-008). Animals were randomly allocated into sham-operated and orchiectomy groups. Each assay was repeated for more than 3 times (3 independent determinations). There were at least 3 animals per group for every determination. Total number of animals used in this study was more than 20 rats.

### Orchiectomy

Orchiectomy was conducted using a castration rat model as described previously [30]. All surgical procedures were performed under sterile conditions and general anesthesia, which was comprised of inhalation of 1%~2% isoflurane. For the orchiectomy, a 10-mm incision was made in the skin of the lower abdomen over one testis of a rat. The testis and associated fat pad were pulled with forceps. The connective tissues leading to the testis which included the fat pad, testicular vein, and vas deferens were tied using surgical silk. Connective tissues between the testis and knot around the connective tissue were cut. Finally, the connective tissues were returned to the body cavity. The wound was cleaned and sutured. In the sham group, animals received a 3-cm wound, which was immediately stitched closed. Four weeks after surgery, rats were euthanized via terminal bleeding under isoflurane-anesthesia, and femurs and tibias were collected. Following the removal of adherent soft tissues, bone tissues were washed with phosphate-buffered saline (PBS, 0.14 M NaCl, 2.6 mM KCl, 8 mM Na_2_HPO4, and 1.5 mM KH_2_PO_4_).

### Quantification of plasma testosterone

Levels of plasma testosterone were quantified according to a previous method [31]. After surgery for 4 weeks, blood was collected from a tail vein of rats. Following centrifugation, the plasma fraction was prepared. Testosterone in plasma was extracted with diethyl ether. The organic phase was evaporated to dryness. Levels of plasma testosterone were measured with a colorimetric competitive enzyme immunoassay kit (Enzo Life Sciences, Farmingdale, NY, USA). Results were read on a colorimetric plate reader (BioTek, Vermont, FL, USA) at 405 nm.

### Analysis of μCT

The bone volume and architecture were evaluated using μCT as described previously [32]. The μCT analysis was conducted using a Skyscan 1176 scanner (Bruker, Billerica, MA, USA). The scanning axis nominally coincided with the diaphyseal axis of the control femur. Femurs with a bone defect were scanned using the same parameters (9 μm per slice, 65 kV, 385 μA, 1-mm Al filter, and a 3300-ms exposure time). High-resolution images of the femurs were generated for 3D polygonal resampling using Skyscan 3D-Creator software (Bruker), and morphometric parameters, including the trabecular number (Tb.N.), trabecular separation (Tb.Sp.), and trabecular thickness (Tb.Th.), were calculated for trabecular bone regions of interest (ROIs) using a Skyscan CT-Analyzer (vers. 1.10, Bruker) as described previously [33]. In addition, the cortical bone area, total area ratio, and cortical bone/total area ratio were analyzed as cortical architectural parameters.

### Test of reduced-platen compression (RPC)

**Fig 1:**
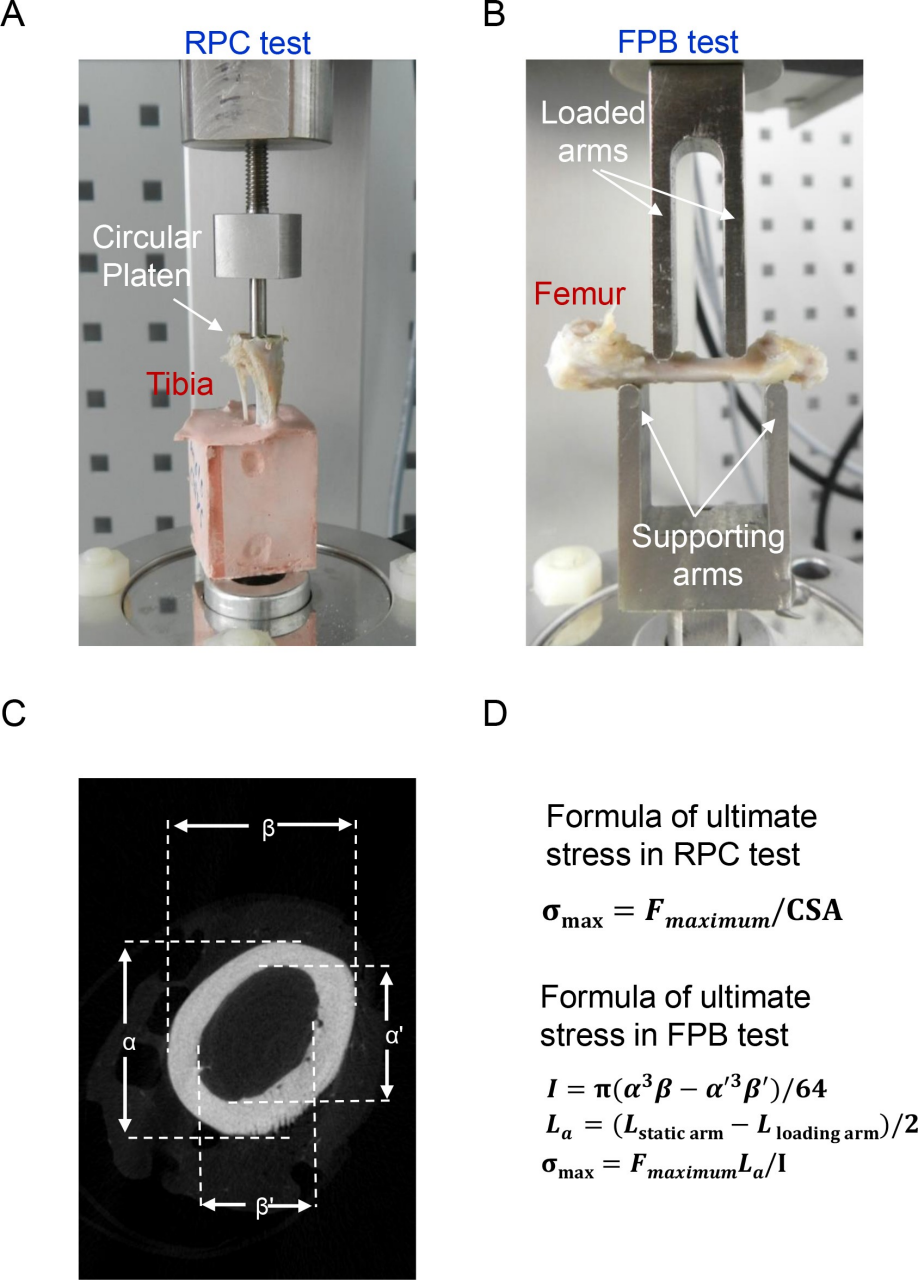
Biomechanical and tomographic methodologies. Male Wistar rats were subjected to sham operation or orchiectomy. After sacrifice, tibias and femurs from sham and orchiectomized rats were collected for analyses of mechanistic reduced-platen compression (RPC) and four-point bending (FPB) (A and B) as well as micro-computed tomography (μCT) (C). In the RPC test, the upper cortical shell of a tibia was removed in order to expose the trabecular region. The strength of trabecular bone was measured using a circular platen to compress the trabecular bone (A). In the FPB test, a femur was placed across two supporting arms and compressed by double loaded arms with a constant force until the specimen broke (B). The external (α and β) and internal (α' and β') diameters of the femur were measured by μCT in order to calculate the cross-sectional area (CSA) (C). The ultimate stress (σ_max_) in the RPC and FPB tests were calculated according to two different formulas (D). *F*_*maximum*_, maximum force during loading history; *I*, area of inertia; L_α_, distance between the supporting and loading pins; *L*_*loading arm*_, length of the loading span; *L*_*static arm*_, length of the supporting span.

The RPC test was performed accorded to a modified protocol following a previously described method [34]. Prior to this mechanical test, tibia specimens from the sham and orchiectomized groups were immersed in 0.9% saline solution for 2 h for rehydration. The upper part of the cortical shell was removed in order to expose the trabecular region of the tibia. The size of the platen was reduced to 2 mm in diameter, and the tibia specimen was aligned such that contact was only made with cancellous bone (Fig 1A). An extensometer (Model 5500, Bose, Framingham, MA, USA) was applied to compress the tibia specimen for 2 mm in depth in the central part. The rate of displacement was 0.5 mm/min. The load and displacement were recoded every 10 ms. The cross-sectional area (CSA) was the area of the reduced platen (3.14 mm^2^). The maximum loading force was divided by the CSA to calculate the ultimate stress (σ_max_) (Fig 1D).

### Test of four-point bending (FPB)

The FPB test was conducted following a modified protocol described previously [35]. Prior to the mechanistic test, a femur was thawed at room temperature and then rehydrated in a 0.9% saline solution for 2 h. An extensometer (Bose) was used to compress the femur specimen for analysis of material resistance and deformation behavior. This apparatus was built of two major parts, the upper loading arms and the lower supporting arms (Fig 1B). The lengths of the upper and lower arms were 10 and 20 mm, respectively. The rate of displacement was 0.5 mm/min. Femur specimens were compressed to 2 mm or until fracture. The load and displacement were recoded every 10 ms. The area moment of inertia (I) was calculated according to the formula shown in Fig 1D as described previously [36]. Considering the hollow structure of the diaphysis, values of α, α’, β, and β’ in the formula were analyzed by μCT images and used to calculate I. Then, the ultimate stress (σ_max_) was calculated according to the formula in Fig 1D. In that formula, F is the maximum load, and L_a_ is the moment arm in the FPB test.

### Statistical analysis

The statistical significance of differences between groups was evaluated using a one-way analysis of variance (ANOVA) with Duncan’s multiple-range test. Differences were considered statistically significant at *p* values of < 0.05. Correlations between tomographic and biomechanical differences in the trabecular bone and cortical bone were evaluated by a linear regression analysis. The *r* value of a linear regression was calculated in order to present the association of those two parameters.

## Results

### Orchiectomy caused significant decreases in testosterone levels

**Fig 2:**
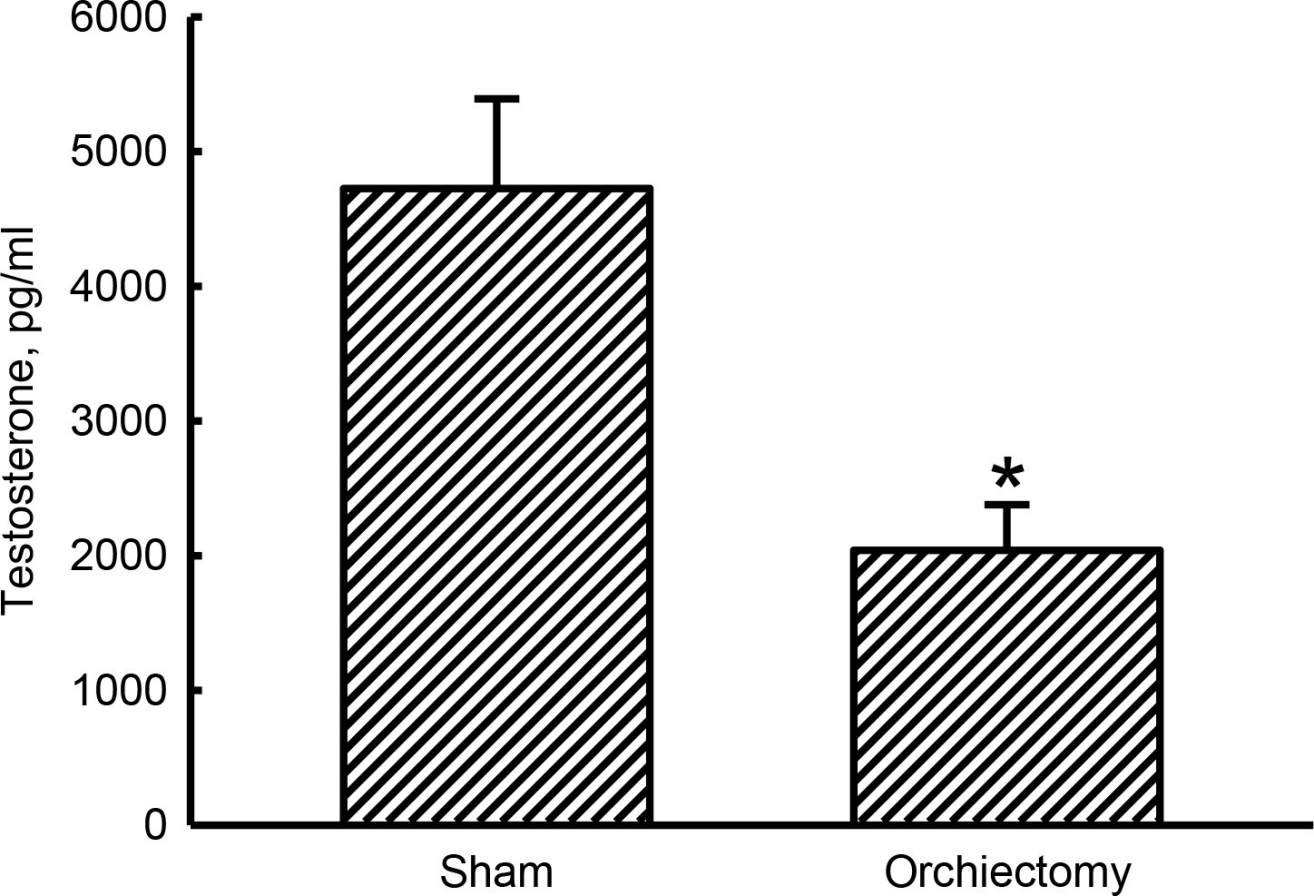
Effects of orchiectomy on reductions in testosterone levels. Male Wistar rats were subjected to sham operation or orchiectomy. Four weeks after surgery, blood samples were collected for analysis of plasma testosterone using an enzyme-linked immunosorbent assay. Each value represents the mean ± SD of three independent determinations. * Indicates that the value significantly (*p* < 0.05) differed from the sham group.

Levels of plasma testosterone in untreated animals were detected (Fig 2). In contrast, at 4 weeks after the orchiectomy, male Wistar rats exhibited a 57% reduction in levels of plasma testosterone compared to the sham group (*p* = 0.0002).

### Analyses of tomographic parameters revealed loss of trabecular bone in orchiectomized rats

**Fig 3:**
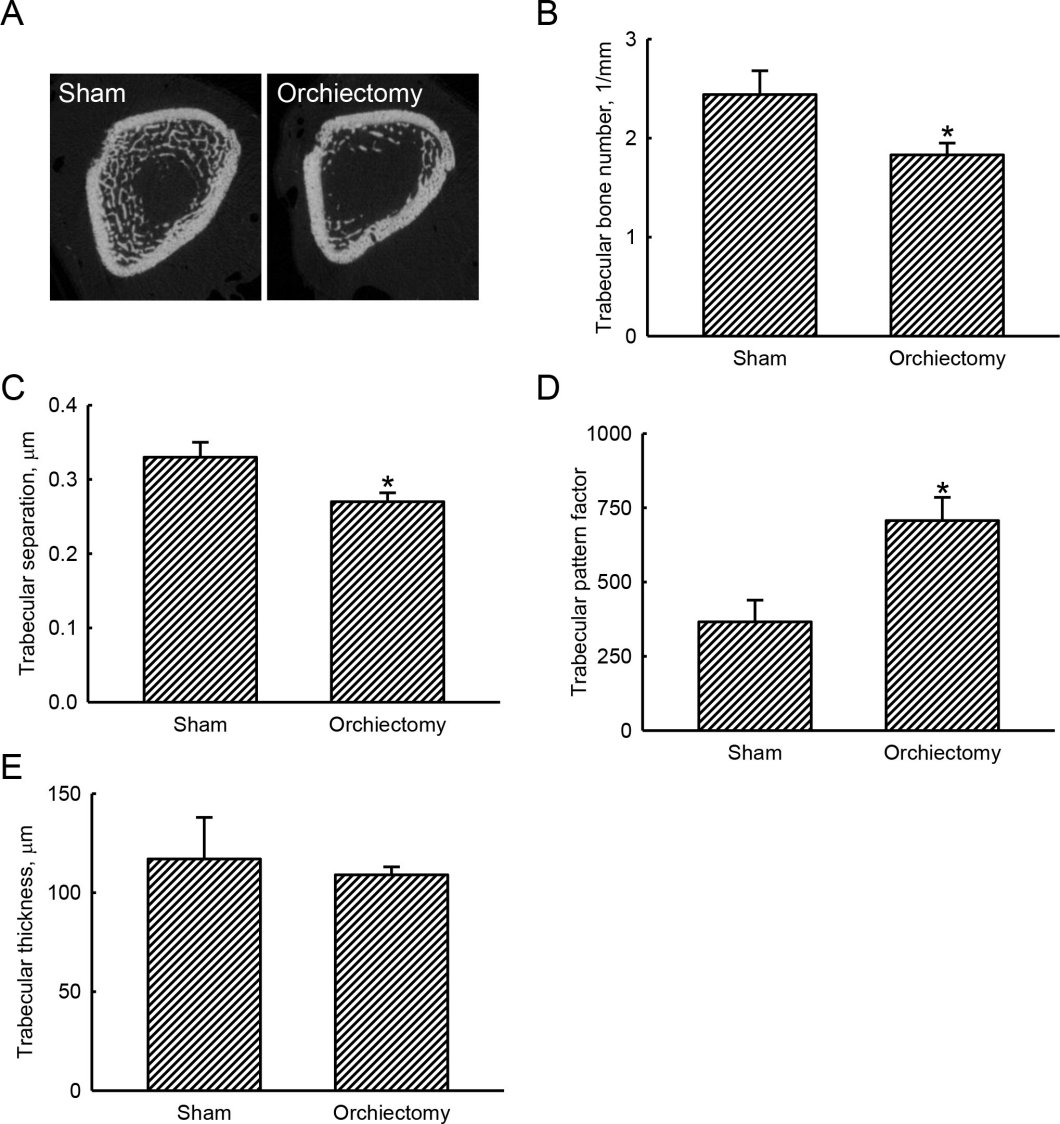
Effects of orchiectomy on tomographic changes of trabecular bone. Male Wistar rats were subjected to sham operation or orchiectomy. Four weeks after surgery, animals from the sham and orchiectomy groups were sacrificed, and their femurs were collected for analysis by micro-computed tomography (μ-CT). The distal femur was scanned with μ-CT, and representative cross-sectional images are presented (A). Tomographic parameters, including the trabecular bone number (B), trabecular separation (C), trabecular pattern factor (D), and trabecular thickness (E), were examined and statistically analyzed. Each value represents the mean ± SD of three independent determinations. * Indicates that the value significantly (*p* < 0.05) differed from the sham group.

Representative tomographic images from scanning femurs using μCT showed that at 4 weeks after the orchiectomy, an obvious decrease in the bone volume and an increase in bone porosity had been induced (Fig 3A). Compared to sham animals, 4 weeks after being castrated, rats showed a significant 25% reduction in the trabecular bone number (*p* = 0.0038) (Fig 3B). At the same time, the orchiectomy caused a decrease in the separation of trabecular bone by 18% compared to sham rats (*p* = 0.021) (Fig 3C). Afterward, the trabecular pattern factor was meaningfully augmented by 93% (*p* = 0.0018) at 4 weeks following the orchiectomy (Fig 3D). In contrast, the trabecular thickness between sham and orchiectomized animals was not influenced (*p* = 0.8512) (Fig 3E).

### Mechanistic analyses by an RPC test further demonstrated the suppressive effects of orchiectomy on the strength of trabecular bone

**Fig 4:**
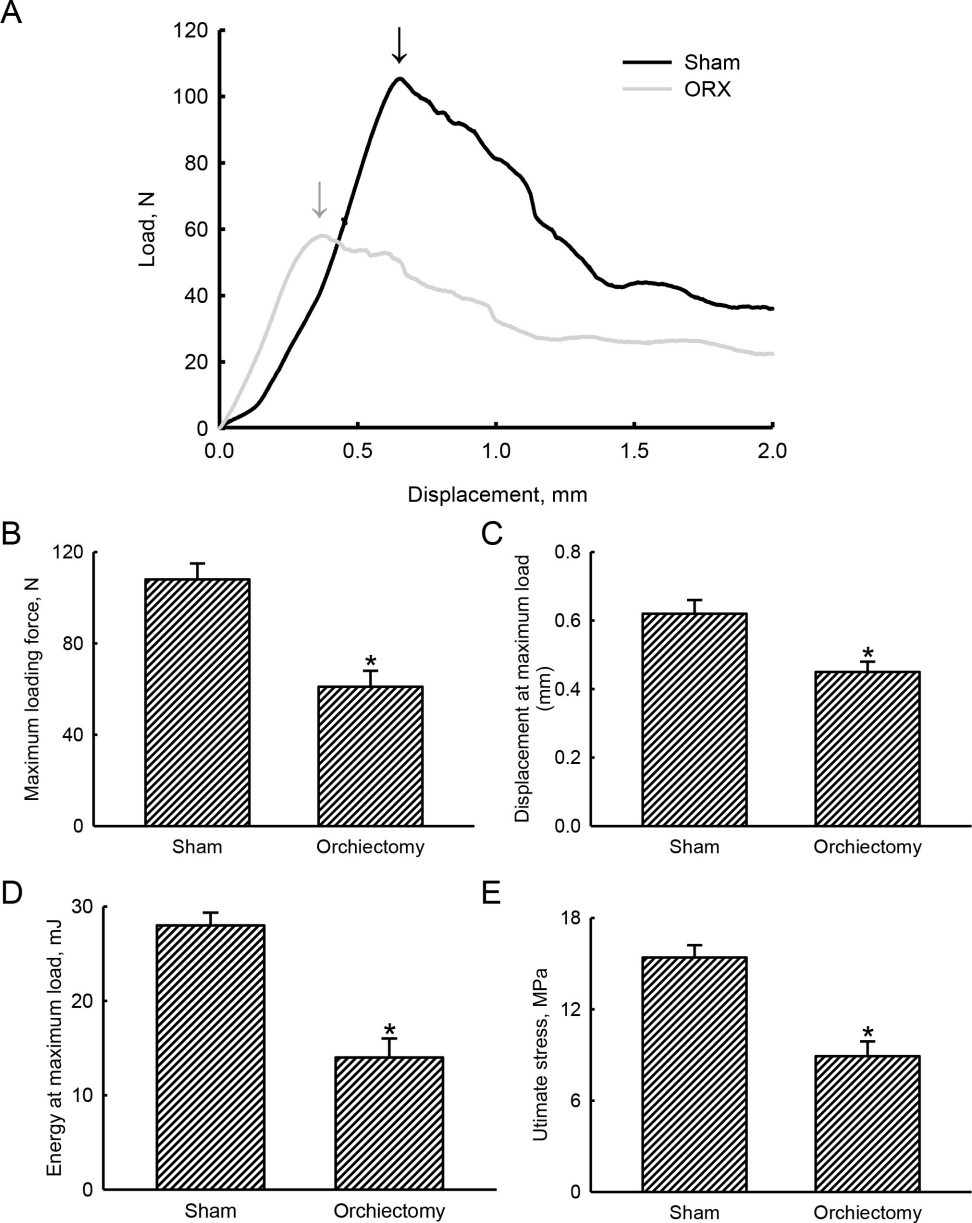
Effects of orchiectomy on biomechanical changes of trabecular bone. Male Wistar rats were subjected to sham operation or orchiectomy. Four weeks after surgery, animals from the sham and orchiectomy groups were sacrificed, and their tibias were collected for analysis of a reduced-platen-compression test. The upper cortical shell of a tibia was removed to expose the trabecular region. A reduced-area platen was used to compress the central area of trabecular bone in the proximal tibia. The compression load and displacement of the tibia were recorded. Representative load-displacement curves in sham and orchiectomized rats are presented (A). Arrows indicate the maximum loading force. Values of these mechanistic parameters, including the maximum loading force (B), displacement at the maximum load (C), energy at the maximum load (D), and ultimate stress (E), were calculated and statistically analyzed. Each value represents the mean ± SD of three independent determinations * Indicates that the value significantly (*p* < 0.05) differed from the sham group.

In the sham group, a load-displacement curve displayed a maximum loading force to break trabecular bone of 105 N at a displacement of 0.64 mm (Fig 4A). In comparison, 4 weeks after being castrated, the maximum loading force to break trabecular bone of an orchiectomized rat was reduced to 58 N at a displacement of 0.33 mm. In addition, compared to sham rats, the load-displacement curve in orchiectomized animals had obviously shifted (Fig 4A). After being orchiectomized, the maximum loading force was diminished by 44% (*p* = 0.0012) (Fig 4B). In contrast, castration led to a 37% drop in the displacement at the maximum load compared to control animals (*p* = 0.0045) (Fig 4C). Fascinatingly, the energy at maximum load to break trabecular bone of sham rats was 28.0 ± 1.4 mJ (Fig 4D). However, the energy at the maximum load to break trabecular bone of orchiectomized rats was reduced by 50% (*p* = 0.0029). Consequently, compared to the sham group, the orchiectomy caused a 42% decrease in the ultimate stress (*p* = 0.0011) (Fig 4E).

### The effects of orchiectomy were additionally confirmed with correlational analyses of our tomographic and biomechanical data

**Fig 5:**
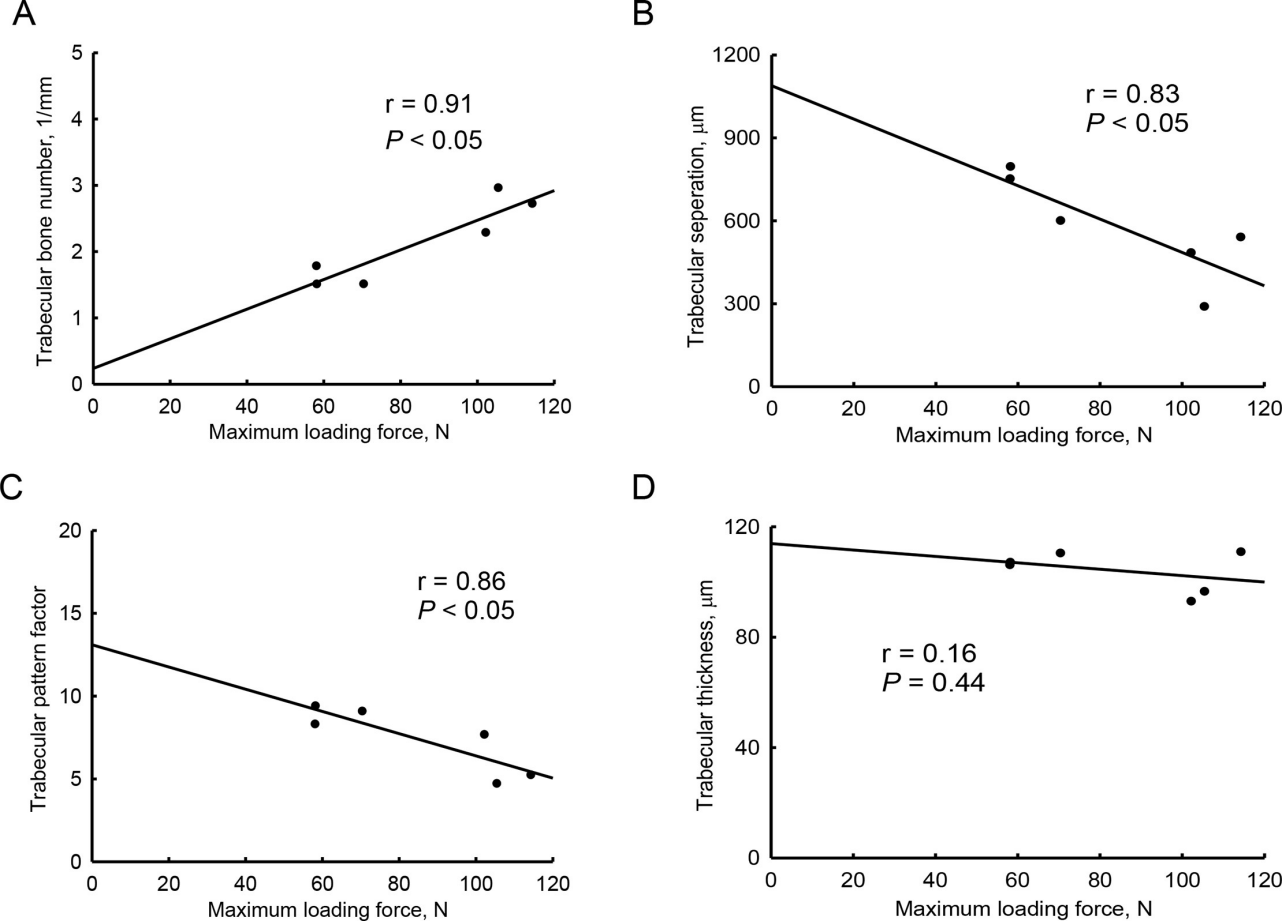
Correlations of the biomechanical maximum loading force with tomographic parameters in orchiectomy-treated trabecular bone. Trabecular bones from sham-operated and orchiectomized rats were analyzed using micro-computed tomography and a reduced-platen compression test. The trabecular bone number, trabecular separation, trabecular pattern factor, trabecular thickness, and maximum loading force were measured. Correlations of the maximum load force with trabecular bone number (A), trabecular separation (B), trabecular pattern factor (C), and trabecular thickness (D) were analyzed using linear regressions.

The castration-induced tomographic decrease in the trabecular bone number was highly correlated with the orchiectomy-induced reduction in the maximum loading force that could break trabecular bone (*r* = 0.91, *p* < 0.05) (Fig 5A). In addition, the tomographic parameters of trabecular separation (*r* = 0.83, *p* < 0.05) and trabecular pattern factor (*r* = 0.86, *p* < 0.05) were exceptionally associated with the maximum loading force in orchiectomized rats (Fig 5B and 5C). However, the tomographic trabecular thickness was not correlated with the biomechanical maximum loading force in the early stage of male osteoporosis (*r* = 0.16, *p* = 0.44) (Fig 5D).

### Orchiectomy did not change tomographic architecture and biomechanical strength of cortical bone

Effects of orchiectomy on tomographic changes in the architecture of cortical bone were determined (Table 1). Four weeks after being castrated, the thickness of cortical bone in male Wistar rats was not affected (*p* > 0.05). In addition, the cortical bone area and total bone section area were not influenced at 4 weeks after the orchiectomy (*p* > 0.05) (Table 1). Consequently, the ratio of the cortical bone area over total bone section area did not change in orchiectomized rats (*p* > 0.05) (Table 1).

**Table 1:** Tomographic analyses of cortical bone.

	Sham	Orchiectomy
**Cortical bone thickness (mm)**	0.313 ± 0.014	0.326 ± 0.019
**Cortical bone area (mm**^**2**^**)**	7.022 ± 0.170	6.948 ± 0.214
**Total bone section area (mm**^**2**^**)**	8.200 ± 0.343	8.052 ± 0.265
**Ratio of cortical area/total section area (%)**	85.90 ± 0.02	86.40 ± 0.02

Male Wistar rats were subjected to sham operation or orchiectomy. Four weeks after surgery, animals were sacrificed, and their femurs were collected. These cortical bones were scanned using μCT. The cortical bone thickness, cortical bone area, and total bone section area were quantified. The ratio of cortical bone area over total bone section area were calculated. Each value represents the mean ± SD of three independent determinations.

Biomechanical analyses using an FBP test were performed in order to determine the effects of orchiectomy on the strength of cortical bone (Table 2). At 4 weeks after being castrated, the biomechanical maximum loading force and displacement at the maximum load of cortical bone were not affected (*p* > 0.05). In orchiectomized rats, the biomechanical stiffness and energy at the maximum load did not change (*p* > 0.05) compared to sham animals (Table 2). Moreover, orchiectomy in male rats did not influence the biomechanical ultimate stress (*p* > 0.05) (Table 2).

**Table 2:** Mechanical analysis of cortical bone.

	Sham	ORX
**Maximum loading force (N)**	177 ± 12	178 ± 8
**Displacement at maximum load (mm)**	0.85 ± 0.29	0.86 ± 0.29
**Bone stiffness (N/mm)**	248 ± 63	225 ± 34
**Energy at maximum load (mJ)**	79 ± 23	91 ± 26
**Ultimate stress (MPa)**	74 ± 9	86 ± 9

Male Wistar rats were subjected to sham operation or orchiectomy (ORX). Four weeks after surgery, animals were sacrificed, and their femurs were collected. Mechanical analyses of these cortical bones were carried out using a four-point-bending test. The maximum force, displacement at the maximum force, stiffness, energy of the maximum force, and ultimate stress were calculated and statistically analyzed. Each value represents the mean ± SD of three independent determinations.

**Fig 6:**
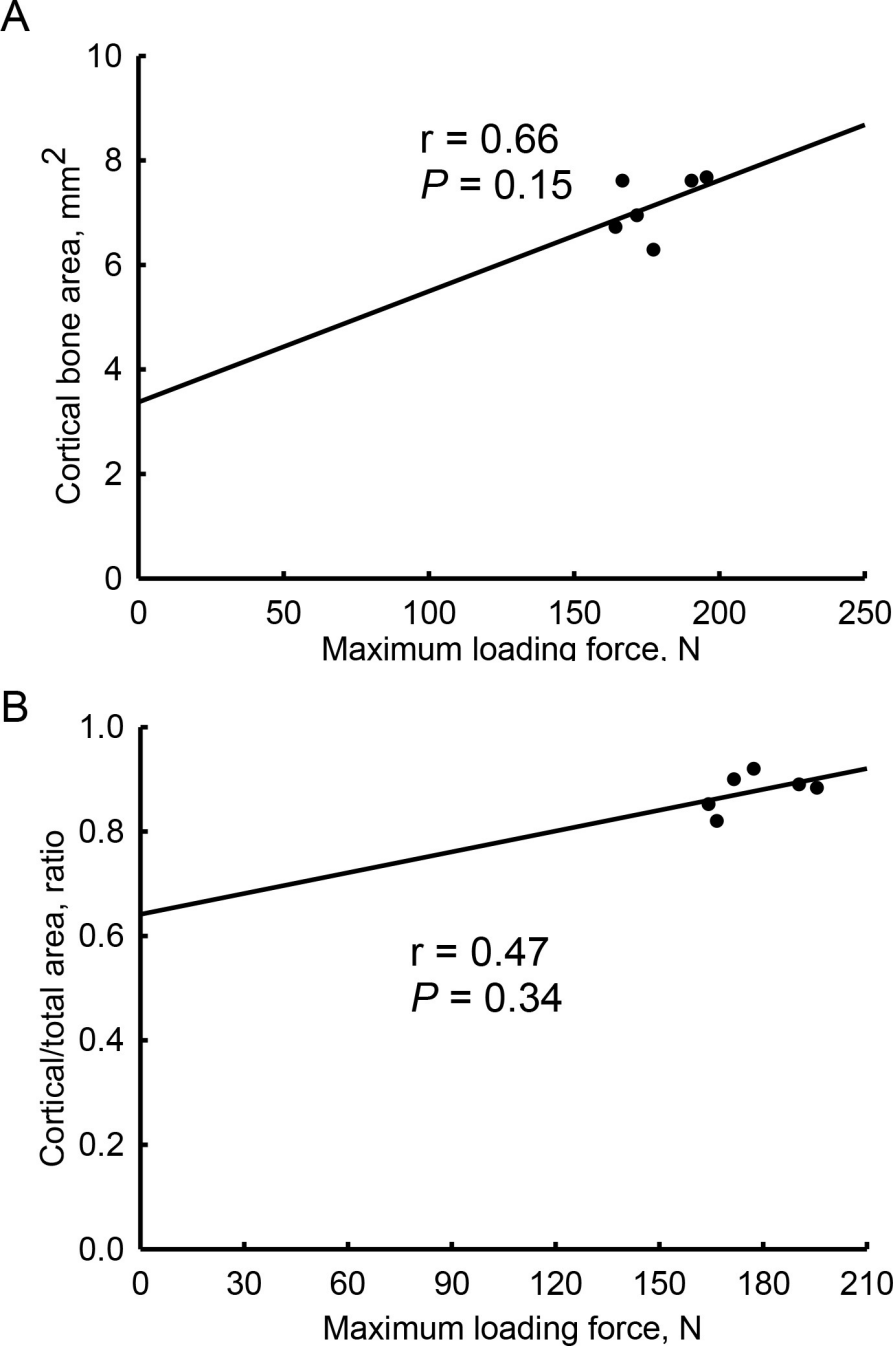
Associations of the biomechanical maximum loading force with tomographic parameters in orchiectomy-treated cortical bone. Cortical bones from sham-operated and orchiectomized rats were analyzed using micro-computed tomography and a reduced-platen compression test. The tomographic cortical bone area, ratio of cortical bone area over the total bone section area, and mechanical maximum loading force were measured. Correlations of the mechanical maximum loading force with the tomographic cortical bone area (A) and ratio of cortical bone area over total bone section area (B) were analyzed using linear regressions.

Correlations of the biomechanical maximum loading force with tomographic parameters in cortical bone were examined (Fig 6). In orchiectomized rats, the tomographic cortical bone area was not significantly correlated with the biomechanical maximum loading force in cortical bone (*r* = 0.66, *p* = 0.15) (Fig 6A). The ratio of the cortical bone area over total bone section area also showed a medium association with the biomechanical maximum loading force (*r* = 0.47, *p* = 0.34) (Fig 6B).

## Discussion

The present study provides tomographic and biomechanical evidence to show noteworthy differences in the microarchitecture and strength of trabecular bone in the early stage of male osteoporosis. Orchiectomy is a valuable experimental model for research of osteoporosis and osteoporotic fractures [30]. This study showed that levels of testosterone in plasma of male Wistar rats were meaningfully decreased at 4 weeks following orchiectomy. Testosterone, a male sex hormone with the highest concentration, can control the development and maintenance of masculine characteristics [11]. In a human epidemiological study, testosterone was demonstrated to be positively related to the BMD and fracture risk [12]. Separately, our μCT examination showed that 4 weeks after castrating male rats, their bone volume decreased and the bone porosity increased. Accordingly, 1 month after orchiectomy, bone loss similar to that in the early stage of osteoporosis had been induced. Furthermore, our tomographic and biomechanical data demonstrated decrements in the microarchitecture and strength of trabecular bone in orchiectomized rats. In contrast, castration did not affect the tomographic or biomechanical characteristics of cortical bone. Our previous study demonstrated that a bilateral ovariectomy in rats lessened blood 17β-estradiol levels, the bone torsion force and bone mineral contents [27]. Vorland et al. reported that subjection of rats to ovariectomy led to a noteworthy loss of trabecular bone but did not affect cortical bone [37]. As the mechanism, the ovariectomy-induced estrogen deficiency induces bone loss due to a significant augmentation in osteoclast numbers [26]. In a male animal model of osteoporosis, orchiectomy could augment bone turnover and triggered bone loss via activation of the RANKL-OPG signaling pathway [26, 29]. In men, loss of trabecular bone occurs much earlier than harm to cortical bone [8]. Herein, we present tomographic and biomechanical evidence to show specific loss of trabecular bone in the early stage of male osteoporosis.

Orchiectomy interrupted the microarchitecture of trabecular bone. μCT radiographic studies showed that 4 weeks after orchiectomy in male Wistar rats, the volume of trabecular bone had diminished. The bone volume fraction can directly elucidate osteoporosis-induced variations in the assembly and strength of cancellous bone [38]. Subsequently, the porosity of cancellous bone increased following castration. In addition, our tomographic data displayed orchiectomy-induced reduction in the trabecular bone number. A diminished trabecular bone number can clarify deterioration of the spongy bone microarchitecture and consequent existence of bone loss [39]. In addition, treatment of male Wistar rats with orchiectomy decreased the separation of trabecular bone. Weinstein and Hutson stated that a reduction in the trabecular width was directly connected to bone loss with aging [34]. In comparison, the trabecular pattern factor was enhanced after orchiectomy. The trabecular pattern factor is a histomorphometric parameter for informal quantification of the bone microarchitecture [40]. An increase in the value of the trabecular bone factor characterizes weaker trabecular connectivity [41]. Our previous study demonstrated the benefits of chitosan nanofiber scaffolds in reducing the trabecular bone factor value to improve bone healing [32]. Recently, we further showed a relationship of the trabecular bone factor with fracture healing [33]. A previous study reported architectural changes in trabecular bone after rats had been castrated for 3~4 months [42]. Our current analyses of tomographic images and parameters disclosed the effects of orchiectomy on altering the microarchitecture of trabecular bone in the primary phase of male osteoporosis.

Our biomechanical analysis additionally verified the orchiectomy-induced reduction in the strength of trabecular bone. In this study, a reduced-platen compression test was applied to measure the strength of trabecular bone in tibias. This apparatus compresses the tibial specimen until a deforming point, which directly reflects the material resistance [34, 43]. After the orchiectomy, the maximum loading force to break trabecular bone had significantly decreased. The displacement at the maximum load was also reduced in castrated rats. Moreover, the energy that was necessary to expand the maximum loading force to break a tibia decreased in castrated animals compared to the sham group. In a biomechanical test, decreases in the maximum loading force, displacement, and energy indicated a lessening of the strength of trabecular bone. Remarkably, the ultimate stress of trabecular bone in tibias declined following castration. The ultimate stress, also known as the compressive strength, is the capacity of bone to withstands loads [44]. A diminution in the ultimate stress of cancellous bone indicates orchiectomy-induced weakening of the bone strength. More importantly, our biomechanical data representing the maximum loading force illustrated high correlations with the tomographic trabecular bone number, trabecular separation, and trabecular pattern factor in castrated rats. Previous studies reported that radiographic BMD cannot accurately reflect the osteoporosis-associated fracture risk [21–23]. Our present study additionally provides reliable compression data to support orchiectomy-triggered disruptions in the microarchitecture and strength of trabecular bone in the early stage of male osteoporosis.

Orchiectomy did not affect the microarchitecture or strength of cortical bone. Our radiographic data revealed that after castration, the thickness and bone area fraction of cortical bone in male Wistar rats were not influenced compared to sham animals. Our present findings are similar to the work of Blouin et al. who described how at 1 month after an orchietomy, the architecture of cortical bone in aged male Wistar rats had not changed [42]. Vandenput et al. showed a considerable difference in the volume of cortical bone 4 months after orchiectomy in aged male Wistar Hannover rats [45]. Another study also revealed that long term (> 4 months) after orchiectomy, the cortical bone mass was significantly affected [46]. In women, trabecular bone is lost with age [10]. In contrast, loss of cortical bone begins after the age of 75 years in men [8]. Nonetheless, loss of cortical bone in women occurs much earlier than in men [9]. In parallel, 4 weeks after orchiectomy in male Wistar rats, the maximum loading force, displacement at the maximum load, bone stiffness, energy at the maximum load, and ultimate stress of cortical bone were not affected. Recently, Mohamad et al. reported that administration of rats with orchiectomy for 3 months induced more bone loss in trabecular bone than in cortical bone [47]. In comparison, they conducted a three-point bending test but did not find any significant changes in biomechanical strength. Our biomechanical RPC and FPB analyses provide more lines of evidence to verify that compared to cortical bone, orchiectomy can change the microarchitecture or strength of trabecular bone in the early stage of osteoporosis.

## Conclusions

In summary, this study showed that 4 weeks after orchiectomy in male Wistar rats, levels of testosterone in plasma significantly decreased which concurrently prompted bone loss. Our tomographic analyses revealed that orchiectomy caused substantial alterations in the trabecular bone number, trabecular separation, and trabecular pattern factor. Fascinatingly, these orchiectomy-induced tomographic differences in the microarchitecture of trabecular bone in castrated rats were highly associated with biomechanical reductions in the maximum loading force, displacement at the maximum load, energy at the maximum load of trabecular bone. In contrast, there were no significant differences in the tomographic microarchitecture or biomechanical strength of cortical bone 4 weeks after orchiectomy in Wistar rats. Taken together, the present study provides tomographic and biomechanical evidence to show specific insults to the microarchitecture and strength of trabecular bone in the early stage of male osteoporosis.

## Supporting information

S1 Table Raw data for Table 1. (PDF)

S2 Table Raw data for Table 2. (PDF)

S1 Fig Raw data for Fig 2. (PDF)

S2 Fig Raw data for Fig 3. (PDF)

S3 Fig Raw data for Fig 4. (PDF)
