# Supplementary material for: Biomechanical and tomographic differences in the microarchitecture and strength of trabecular and cortical bone in the early stage of male osteoporosis
Source: PLoS One. 2019 Aug 8;14(8):e0219718. doi: 10.1371/journal.pone.0219718 (PMC6687113; doi:10.1371/journal.pone.0219718)
Supplement: S1 Table — (PDF) [file pone.0219718.s001.pdf]

Table 1

	Cortical bone thickness			Cortical bone area			Total bone section area			Total bone section area	
	Sham	ORX		Sham	ORX		Sham	ORX		Sham	ORX
1	0.33	0.302	1	7.154	7.227	1	7.757	8.052	1	85.92	86.4
2	0.295	0.33	2	7.17	6.744	2	8.543	7.754	2	85.82	86.38
3	0.314	0.344	3	7.122	6.88	3	8.53	8.217	3	85.91	86.23
4	0.313	0.325	4	7.039	6.753	4	8.2	7.852	4	85.9	86.39
5	0.329	0.341	5	6.852	7.15	5	8.21	8.05	5	85.88	86.4
6	0.305	0.306	6	6.77	7.175	6	7.897	8.252	6	85.98	86.4
7	0.325	0.35	7	7.211	6.94	7	8.64	7.887	7	85.88	86.45
8	0.295	0.308	8	6.86	6.71	8	7.86	8.35	8	85.9	86.36
