# Supplementary material for: Biomechanical and tomographic differences in the microarchitecture and strength of trabecular and cortical bone in the early stage of male osteoporosis
Source: PLoS One. 2019 Aug 8;14(8):e0219718. doi: 10.1371/journal.pone.0219718 (PMC6687113; doi:10.1371/journal.pone.0219718)
Supplement: S2 Table — (PDF) [file pone.0219718.s002.pdf]

Table 2

	Maximum loading force			Displascement at maximum load			Bone stiffness			Energy at maximum load			Ultimate stress	
	Sham	ORX		Sham	ORX		Sham	ORX		Sham	ORX		Sham	ORX
1	190	178	1	0.54	0.86	1	315	97	1	105	91	1	65	86
2	162	163	2	1.2	0.56	2	321	115	2	55	119	2	84	94
3	177	162	3	0.85	1.16	3	248	59	3	98	65	3	83	78
4	164	178	4	0.86	1.21	4	179	66	4	64	121	4	74	84
5	192	192	5	0.52	0.51	5	250	85	5	57	59	5	64	99
6	177	186	6	1.15	0.86	6	172	121	6	96	90	6	75	77
