# Supplementary material for: Biomechanical and tomographic differences in the microarchitecture and strength of trabecular and cortical bone in the early stage of male osteoporosis
Source: PLoS One. 2019 Aug 8;14(8):e0219718. doi: 10.1371/journal.pone.0219718 (PMC6687113; doi:10.1371/journal.pone.0219718)
Supplement: S1 Fig — (PDF) [file pone.0219718.s003.pdf]

Fig. 2

	Testosterone	
	Sham	ORX
1	4722	2035
2	4004	2381
3	5450	1713
4	4002	1637
5	5452	2407
6	4700	2051
