# Supplementary material for: Biomechanical and tomographic differences in the microarchitecture and strength of trabecular and cortical bone in the early stage of male osteoporosis
Source: PLoS One. 2019 Aug 8;14(8):e0219718. doi: 10.1371/journal.pone.0219718 (PMC6687113; doi:10.1371/journal.pone.0219718)
Supplement: S2 Fig — (PDF) [file pone.0219718.s004.pdf]

Fig. 3B

Trabecular bone number

	Sham	ORX
1	2.51	1.8
2	2.7	1.64
3	2.65	2.04
4	2.75	1.72
5	2.16	1.96
6	2.26	1.84

Fig. 3C

Trabecular separation

	Sham	ORX
1	0.303	0.279
2	0.351	0.269
3	0.327	0.259
4	0.357	0.268
5	0.297	0.277
6	0.325	0.261

Fig. 3D

Trabecular pattern factor

	Sham	ORX
1	35	71
2	31	81
3	37	61
4	43	78
5	28	64
6	46	70

Fig. 3E

Trabecular thickness

	Sham	ORX
1	141	110
2	90	115
3	116	103
4	95	104
5	137	114
6	115	113
