# Supplementary material for: Biomechanical and tomographic differences in the microarchitecture and strength of trabecular and cortical bone in the early stage of male osteoporosis
Source: PLoS One. 2019 Aug 8;14(8):e0219718. doi: 10.1371/journal.pone.0219718 (PMC6687113; doi:10.1371/journal.pone.0219718)
Supplement: S3 Fig — (PDF) [file pone.0219718.s005.pdf]

Fig. 4B

	Maximum loading force	
	Sham	ORX
1	108	61
2	101	68
3	115	54
4	116	53
5	102	60
6	105	69

Fig. 4C

	Displacement at maximum load	
	Sham	ORX
1	3.83	2.55
2	3.5	2.95
3	4	2.75
4	4.05	3.05
5	3.4	2.55
6	3.7	2.65

Fig. 4D

	Energy at maximum load	
	Sham	ORX
1	31	12
2	27	17
3	29	14
4	31	16
5	27	13
6	29	14

Fig. 4E

	Utimate stress	
	Sham	ORX
1	15.1	7.9
2	16	8.8
3	14.7	9.8
4	15.8	9.7
5	14.1	7.8
6	15	9
